# Autism through midlife: trajectories of symptoms, behavioral functioning, and health

**DOI:** 10.1186/s11689-023-09505-w

**Published:** 2023-11-03

**Authors:** Jinkuk Hong, Leann Smith DaWalt, Julie Lounds Taylor, Aasma Haider, Marsha Mailick

**Affiliations:** 1 Waisman Center, University of Wisconsin-Madison, 1500 Highland Ave, Madison, WI 53705 USA; 2 Vanderbilt University Medical Center, 1211 Medical Center Drive, Nashville, TN 37232 USA; 3 Lawrence University, 711 E. Boldt Way, Appleton, WI 54911 USA

**Keywords:** Autism in adulthood, Developmental trajectories, Symptoms, Functioning, Health, Midlife and aging, Accelerated longitudinal design

## Abstract

**Background:**

This study describes change in autism symptoms, behavioral functioning, and health measured prospectively over 22 years. Most studies tracking developmental trajectories have focused on autism during childhood, although adulthood is the longest stage of the life course. A robust understanding of how autistic people change through midlife and into older age has yet to be obtained.

**Methods:**

Using an accelerated longitudinal design with 9 waves of data, developmental trajectories were estimated from adolescence through midlife and into early old age in a community-based cohort (*n* = 406). The overall aim was to determine whether there were age-related increases or decreases, whether the change was linear or curvilinear, and whether these trajectories differed between those who have ID and those who have average or above-average intellectual functioning. Subsequently, the slopes of the trajectories were evaluated to determine if they differed depending on age when the study began, with the goal of identifying possible cohort effects.

**Results:**

There were significant trajectories of age-related change for all but one of the measures, although different measures manifested different patterns. Most autism symptoms improved through adulthood, while health worsened. An inverted U-shaped curve best described change for repetitive behavior symptoms, activities of daily living, maladaptive behaviors, and social interaction. For these measures, improved functioning was evident from adolescence until midlife. Then change leveled off, with worsening functioning from later midlife into early older age. Additionally, differences between autistic individuals with and without ID were evident. Although those who have ID had poorer levels of functioning, there were some indications that those without ID had accelerating challenges in their aging years that were not evident in those with ID – increases in medications for physical health problems and worsening repetitive behaviors.

**Conclusions:**

Meeting the needs of the increasingly large population of autistic adults in midlife and old age requires a nuanced understanding of life course trajectories across the long stretch of adulthood and across multiple domains. Given the heterogeneity of autism, it will be important not to generalize across sub-groups, for example those who are minimally verbal and those who have above-average intellectual functioning.

**Supplementary Information:**

The online version contains supplementary material available at 10.1186/s11689-023-09505-w.

## Background

Autism is a neurodevelopmental disorder and as such the investigation of developmental trajectories of autism symptoms and other characteristics has long been a critical approach to empirical research. Studies using repeated measures over a period of months (e.g., [1–4]) or years [5, 6] are seen as the “gold standard” method for tracing these trajectories, and such studies have offered significant insights into brain and behavioral development in autism. Although most studies tracking developmental trajectories in those diagnosed with autism have focused on childhood, there are now a number of studies that extend the trajectories into the transition years and young adulthood [7–12]. Adulthood is the longest stage of the life course, yet until recently it has not been the focus of much longitudinal research. Thus, a robust understanding of how the features of autism change through adulthood and into older age has yet to be obtained.

To address this gap in the literature, the current study investigated trajectories of autism symptoms, behavioral functioning, and health from adolescence through midlife and into the early years of old age. We focused on autism symptoms and behavioral functioning as these are key features of autism and because their trajectories through childhood and into early adulthood are relatively well-established. We focused on physical health given its importance for quality of life in adulthood [13]. Physical health during adulthood also has been identified as a research priority by autistic adults and their families (e.g., [14, 15]).

One focus of past research on autism in adulthood has been on childhood predictors of adult outcomes such as employment, independent living, and social integration, revealing the importance of cognitive ability and language development as predictors of some but not all adult outcomes [10, 14, 16, 17]. Data from these studies also have been used to track change from childhood primarily up to the early adult years, highlighting how adaptive skills develop and maladaptive behaviors diminish when compared with these domains during childhood [7].

Virtually absent from the autism research literature is concomitant tracking of change from early adulthood into midlife and beyond, which is the focus of the present research.

Notably, most past research focused on the adolescence-to-adult transition suggests that improvements in skills and behaviors do not progress in a linear fashion. Autism symptoms and maladaptive behaviors have generally been found to improve across childhood and adolescence and into early adulthood ([18, 19], but for an exception see [10]). However, these improvements in symptoms and behaviors slow after youth with autism exit school, with improvement even stopping for some young adults [20]. Daily living skills tend to improve throughout adolescence and early adulthood, but plateau when adults with autism are in their mid-to-late 20 s and then begin to decline [8, 21]. However, little is known about the trajectories of these domains in the later decades of life.

Health is another domain that is important to consider when examining trajectories into older age. Studies examining changes in health over time for autistic adults have found that, for most individuals, body mass index and prescription medication use increase throughout early adulthood [22, 23]. Tracing changes in health and behavioral functioning among autistic adults as they move into midlife and early old age may improve understanding of earlier mortality [24–26] and could point toward strategies for reducing this disparity.

Notably, beyond longitudinal studies that extend from childhood into adulthood, most studies of autistic adults have included primarily or exclusively individuals who do not have intellectual disability (ID) (e.g., [27–29]). Although recent epidemiological evidence estimates that about 38% of autistic children are classified as having ID [30], the current population of autistic adults who have ID is even larger. Therefore, understanding midlife and old age in both those with ID and those with average or above average intellectual functioning is of vital importance. Although there are other factors that contribute to the heterogeneity of autism, ID status stands out as particularly significant in shaping the emergence of symptoms during childhood and that continues to be prominent in adulthood.

### Present study

The present study describes changes in autism symptoms, behavioral functioning, and health measured prospectively over 22 years. Using an accelerated longitudinal design approach to statistical analysis, we estimate developmental trajectories of change extending from adolescence through midlife and into the early years of old age in a community-based cohort that includes both autistic individuals with ID and those who have average or above-average intellectual functioning. We aim to identify points during the life course, starting in adolescence, when vulnerabilities may be increasing or decreasing, with a particular focus on midlife, a life stage that has received minimal attention in the autism research literature.

Midlife is a period of the life course that is not precisely defined, although it is often characterized in the general population as the period between 40 and 60 years of age, plus or minus 10 years [31]. It is seen as a stage of life that is a “pivotal period in the life course in terms of balancing growth and decline, linking earlier and later periods of life” [32]. Here we present repeated measures of autism symptoms, behavioral functioning, and health to characterize this pivotal period. Our overall aim is to characterize the patterns of age-related change. Accordingly, we investigated whether each domain showed age-related increases or decreases (or did not change), and whether the change was linear or curvilinear.

Given the heterogeneity of autism, we also evaluated whether these trajectories differed between those who have ID and those who have average or above-average intellectual functioning. Subsequently, we explored whether the slope of the trajectories differed depending on the age of the participant when the study began, with the goal of identifying possible cohort effects.

## Methods

### Data and sample

This report is based on an analysis of data from an ongoing longitudinal study of families of autistic adolescents and adults [33]. The study began in 1998 with families of 406 adolescents and adults diagnosed with autism living in Massachusetts and Wisconsin, and to date it has extended over 22 years. All participating families initially met three inclusion criteria: (1) the family had a son or daughter with an autism diagnosis given by an educational or health professional, (2) the proband was age 10 or older, (3) a researcher-administered Autism Diagnostic Interview-Revised (ADI-R) [34] profile was consistent with the diagnosis. When the study began, almost all (94.6%) met criteria for a diagnosis of autistic disorder. The remaining 22 cases (5.4%) were determined to have ADI-R profiles consistent with a diagnosis of Asperger’s disorder or Pervasive Developmental Disorder-Not Otherwise Specified (PDD-NOS) (see [35]), diagnoses in use at that time. The present analysis used repeated measures that were collected over nine study waves from the primary caregivers, mostly (96%) mothers, via in-home interviews as well as self-administered questionnaires. Although some autistic individuals directly provided data at three of the waves of the study, in the interest of including data from those of all cognitive and communicative ability levels, we use parent-report data for this analysis.

The nine waves of data (here referred to as Time 1 through Time 9) spanned 22 years. Previous reports of analyses from this study reported on shorter spans of time, and were based on fewer rounds of data collection gathered earlier in the life course (e.g., [8, 19, 36]). Although all of the measures reported here were used in our previous reports, here we nearly double the duration of the study period and add three additional repeated measures (Times 6 through Time 9), extending most measures through 2022. Table 1 shows the timing of each wave of data collection along with average ages and age ranges of the probands at each wave. As shown in Table 1, on average, most waves of data collection were approximately 18 months apart except for Time 4 and 5 (about 44 months) and Time 8 and 9 (about 8 years). The average number of study waves for which participants contributed data was six, and more than a half (52.2%) participated in seven or more study waves. Although attrition remains a limitation of this study, those who were lost to attrition did not differ from those who remained in the study through Time 9 with respect to sex, ID status, and the outcome variables. Those who were lost to attrition were an older age at Time 1 (23.1 vs. 19.2 years, *p* < 0.001) than those who remained.

**Table 1:** Waves, dates of data collection, and ages of autistic adolescents and adults

Waves of Data Collection (n)	Dates of Data Collection	Age of autistic adolescents and adults^a^
Time 1 (406)	1988 – 2000	21.9 (9.4) [10.1, 52.1]
Time 2 (360)	2000 –2002	23.6 (9.6) [11.3, 53.9]
Time 3 (344)	2002 – 2003	24.9 (9.4) [12.7, 52.4]
Time 4 (307)	2004 – 2005	26.3 (9.3) [14.6, 53.3]
Time 5 (249)	2007 –2009	29.7 (9.0) [18.3, 57.1]
Time 6 (235)	2009 –2010	31.1 (8.8) [19.8, 58.8]
Time 7 (212)	2011 –2012	32.3 (8.0) [21.9, 60.3]
Time 8 (193)	2013 – 2014	34.2 (8.4) [23.7, 60.5]
Time 9 (146)	2021 – 2022	42.4 (8.6) [31.4, 68.9]

^a^Means, standard deviations (in parentheses), and ranges (in brackets) are presented

The analytic sample consisted of all 406 autistic individuals. Their average age at Time 1 was 21.9 (SD = 9.4), ranging from 10 to 52. The majority were males (73.2%) and over two-thirds (69.7%) had a co-occurring diagnosis of ID. Nearly two-thirds (65.0%) lived with their mothers (and often other family members) at Time 1. During the study, 28 autistic individuals died. The data describing these individuals prior to death are included in the analysis. A larger number of mothers died during the study period (*n* = 73), resulting in change of reporters from the mother to another family member or in a few cases to a family friend. We conducted a sensitivity analysis including only those cases where the mother was the source of data. It revealed that all of the results presented below were fully replicated (see Supplemental Material), reflecting the selection of measures that were well-validated and more objective than subjective reports of life course patterns.

Although the great majority of the families of the autistic individuals were White non-Hispanic (92.6%), there was significant socioeconomic heterogeneity. Median annual household income at Time 1 was between $50,000 and $60,000. Notably, 11.5% earned less than $20,000 per year, when the US poverty line was $17,050 for a family of four (Federal Register, Vol. 65, No. 31, Tuesday, February 15, 2000). Fewer than half of the mothers had achieved a bachelor’s degree (45.1%), and fully one-quarter (26.8%) had no education beyond high school.

### Measures

Bivariate correlations among the measures used in the present research, as well as their associations with age, are presented in Table 2. Although there were significant associations among the measures *within* a given domain, the associations *across* domains were generally non-significant to moderate.

**Table 2:** Bivariate Correlations of Study Variables at Time 1

	ADI-R Social	ADI-R verbal	ADI-R Non-verbal	ADI-R Behavior	WADL	SIB-R	Times with friends	Self-Rated Health	# Psy meds	# non-Psy meds	Adult’s age
ADI-R Social	1.000										
ADI-R verbal	0.568***	1.000									
ADI-R Non-verbal	0.467***	0.755***	1.000								
ADI-R Behavior	0.129*	0.307***	0.025	1.000							
WADL	-0.402***	-0.280***	-0.239***	-0.059	1.000						
SIB-R	0.167**	0.159**	-0.013	0.303***	-0.305***	1.000					
Times with friends	-0.279***	-0.116	-0.114*	-0.024	0.279***	-0.081	1.000				
SRH	-0.065	-0.023	0.043	-0.102*	0.033	-0.117*	0.012	1.000			
# Psy meds	0.075	0.012	-0.019	0.091	-0.110*	0.212***	-0.142**	-0.093	1.000		
# non-Psy meds	0.067	0.047	0.047	-0.023	-0.198***	0.076	-0.040	-0.115*	0.197***	1.000	
Adult’s age	0.200***	0.162**	0.244***	-0.156**	0.158**	-0.278***	0.023	-0.052	0.081	0.113*	1.000

*ADI-R* Autism Diagnostic Interview-Revised, *W-ADL* Waisman Activities of Daily Living Scale, *SIB-R* Scales of Independent Behaviors-Revised. *SRH* Self -Rated Health, *Psy* Psychotropic

^*^ *p* < .05; ** *p* < .01; *** *p* < .001

Measures of autism symptoms included impairments in social reciprocity, impairments in verbal and non-verbal communication, and repetitive behaviors. Measures of behavioral functioning included independence in activities of daily living, maladaptive behaviors, and social participation. Measures of health included ratings of health, number of psychotropic medications prescribed for mental health symptoms, and number of non-psychotropic medications prescribed for physical health symptoms. All measures have been shown to be sensitive to change in prior research [19, 23, 36–38]. Not all measures were obtained at all nine timepoints, but all were collected at least six times over the study period. The specific times of data collection for each measure are indicated below.

#### Measures of autism symptoms

Autism symptoms were assessed using the Autism Diagnostic Interview-Revised (ADI-R) [34] at Time 1 through Time 6. The ADI-R is a standardized diagnostic interview administered to a parent or primary caregiver and used to diagnose autism based on a specified subset of 37 items that constitute a validated algorithm. Our study administered these 37 items at Time 1 to confirm diagnostic status. At each subsequent point of data collection, we administered the 33 items from the core diagnostic algorithm that are applicable to adolescents and adults (4 of the 37 items are specific to childhood). Ratings of current functioning were made at each point of data collection by interviewers who had participated in an approved ADI-R training program. We created four ADI-R sub-scales using 32 of the 33 items based on consultation with one of the instrument’s designers (C. Lord). This grouping of items is based on the clustering of items established by the ADI-R scoring protocol [34], our prior work using this instrument [39], and analysis of the factor structure of the instrument [40]. The sub-scales were impairments in social reciprocity, impairments in verbal communication, impairments in non-verbal communication, and repetitive behavior. A code of 0 signifies the absence of a given symptom, while codes of 1 and 2 indicate impairments characteristic of autism. Some items also used codes of 3, but these are routinely recoded as 2 s [34]. Algorithm items were summed to create the four domain scores. ADI-R items reflecting current levels of impairments in verbal communication were assessed for those individuals who were able to communicate verbally using at least three-word phrases on a daily basis (ADI-R item 30, *n* = 318), 19 of whom shifted from being classified as non-verbal to using three-word phrases on a daily basis during the study period. The ADI-R has demonstrated good test–retest reliability and validity in past research [34, 41]. In this sample, the internal consistency coefficients (Cronbach’s α) at Time 1 were 0.84, 0.71 and 0.53 for social reciprocity, impairments in non-verbal communication, and repetitive behavior, respectively.

#### Measures of behavioral functioning

##### Independence in Activities of Daily Living (ADL)

Independence in activities of daily living was measured longitudinally at seven times of data collection (Time 1, Time 4—Time 9) using the Waisman Activities of Daily Living Scale (W-ADL) [37]. Mothers rated the level of independence of their son or daughter with regards to 17 activities of daily living, measuring performance of personal hygiene (e.g., washing/bathing, grooming, toileting), housekeeping (e.g., home repairs, laundry), meal preparation (e.g., preparing simple food, drinking from a cup, washing dishes), and financial management (banking and managing daily finances) on a scale of 0 to 2 (0 = does not do at all, 1 = does it with help, 2 = does independently). Item scores were summed into a total score with higher scores signifying greater independence in daily living skills. For the present sample, scores ranged from 2 through 34. Past research has shown that the W-ADL is strongly correlated (*r* = 0.82) with the Daily Living scale within the Vineland Screener [8, 37]. The internal consistency (Cronbach’s α) of the W-ADL at Time 1 was 0.903. Criterion validity of the W-ADL for adults with ASD was previously established [37]. The items in the W-ADL span skills generally acquired in early childhood (e.g., drinking from a cup) through those acquired in adulthood (e.g., banking), suggesting good representation of independence in daily living skills across the life course.

##### Maladaptive Behavior

Maladaptive behavior was measured longitudinally at all nine times of data collection using the Behavior Problems subscale of the Scales of Independent Behaviors-Revised (SIB-R) [42]. The SIB-R measures behavior problems, grouped in three domains: internalized behaviors (hurtful to self, unusual or repetitive habits, withdrawal or inattentive behavior), externalized behaviors (hurtful to others, destructive to property, disruptive behavior), and asocial behaviors (socially offensive behavior, uncooperative behavior). If a given behavior problem was manifested during the past 6 months, then frequency (1 = *less than once a month* to 5 = *1 or more times/hour*) and severity (1 = *not serious* to 5 = *extremely serious*) of the behavior were rated by mothers. Standardized algorithms [42] translate the frequency and severity ratings into a General Maladaptive Behavior Index, with higher scores indicating more severe behavior challenges. Reliability and validity have been established by Bruininks et al. [42]. The present analysis uses the General Maladaptive Behavior Index.

##### Social participation

Social participation was assessed longitudinally at all nine times of data collection. At each time point, mothers reported on the frequency with which their son or daughter spent time with friends or neighbors (0 = once a year or never, 1 = several times a year, 2 = once or twice a month, 3 = once a week, 4 = several times a week), an item drawn from the National Survey of Families and Households (www.ssc.wisc.edu/nsfh/).

#### Measures of health

##### Health Ratings

Health ratings were obtained at all nine times of data collection. Mothers rated the health of their son or daughter (1 = poor, 2 = fair, 3 = good, 4 = excellent). Considerable previous research has provided evidence of the validity of such health ratings in predicting mortality [43, 44]. In prior analyses of data from the present study, this measure of health was found to significantly predict mortality over the course of two decades [45].

##### Number of Prescription Medications

As a separate and objective indicator of physical health, at each time point, mothers listed names of all prescription medications currently taken by their son or daughter along with dosage and reason for taking each medication [23]. Medications were separated into psychotropic and non-psychotropic categories. As shown in Table 2, self-rated health was not significantly associated with the number of psychotropic medications, whereas there was a significant negative association between self-rated health and the number of non-psychotropic medications (*r* = -0.115, *p* < 0.05).

*Psychotropic medications* were prescribed for mental health problems and included antipsychotics, antidepressants, anxiolytics and sedative-hypnotics, central nervous system (CNS) stimulants, antimanic medication, anticonvulsant medications that were prescribed to an individual with *no* comorbid diagnosis of epilepsy or seizures (usually for bipolar symptoms), and hypotensive medications that were prescribed to an individual with *no* comorbid diagnosis of hypertension.

*Non-psychotropic medications* were prescribed for physical health problems and included anticonvulsants (for seizures), antiparkinson medications prescribed for side effects of antipsychotic medications (i.e., not prescribed to a person diagnosed with Parkinson's disease), antiemetics, and medications for hypertension, thyroid, diabetes, respiration, hormones, ocular, gastrointestinal (GI), and other miscellaneous purposes. Excluded from our analysis were over-the-counter medications, such as analgesics, laxatives, vitamins, antifungal medication, antacids, and topicals.

Medications were coded and classified based on Physician’s Desk Reference Drug Guide for Mental Health Professionals [46]. These classifications were reviewed and verified by a university-based pharmacist with over 20 years of experience. Separate counts of the number of psychotropic and non-psychotropic prescription medications were used in this report.

#### Predictor variables

The main predictor variable in the present study was age at each study point. Age for each individual was calculated from date of birth to the date of that individual’s data collection at each time point. In addition, we included three other predictors: age at Time 1, sex, and ID status. Age at Time 1 was controlled to evaluate whether the trajectories differed depending on the age of the participant when the study began. This variable was included to provide insight into possible cohort effects in the age-related trajectories (for example, whether individuals who were adolescents at the start of the study showed a different pattern of change as compared to those who were in adulthood when the study began). Sex was coded as 0 = male, 1 = female. ID status (0 = no intellectual disability, 1 = intellectual disability) was determined using a variety of sources. Individuals with standard scores of 70 or below on the Wide Range Intelligence Test (WRIT) [47] and the Vineland Screener [48] were classified as having intellectual disability, consistent with diagnostic guidelines [49]. For individuals with scores above 70 on either measure or when either of the measures for the person was missing, clinical consensus among three psychologists was reached to determine their ID status based on a review of medical and psychological records.

### Data analysis

We used an accelerated longitudinal design (ALD; also referred to as a cohort-sequential design or cross-sequential design) to estimate trajectories in autism symptoms, behavioral functioning, and health of autistic adolescents and adults. The ALD estimates a single long-term longitudinal trajectory by combining multiple short-term longitudinal trajectories of each individual covering different periods. This way, the ALD makes it possible to estimate a growth trajectory spanning wider age ranges than the duration of time covered by a longitudinal study [50]. The present study spanned 22 years, including individuals as young as 10 and as old as 52 when the study began. We are thus able to estimate age-related trajectories spanning approximately 60 years.

The trajectories were estimated for each variable separately in order to evaluate their potentially unique age-related functions. As the present study analyzed data spanning the longest available period during adolescence and adulthood, our goal was to determine *for each indicator* whether there were age-related increases or decreases, whether the change was linear or curvilinear, and whether the trajectories differed between those who have ID and those who have average or above-average intellectual functioning. Subsequently, the slopes of the trajectories were evaluated to determine if they differed depending on age when the study began, with the goal of identifying possible cohort effects.

To assess linear and quadratic trajectories of each dependent variable, we estimated the mixed-effects growth curve models with polynomial functions of age. The Level-1 equation of the quadratic growth model is:

$${Y}_{it} = {\pi }_{0i} + {\pi }_{1i}({a}_{it}-10) + {{\pi }_{2i}({a}_{it} - 10)}^{2} + {e}_{it},$$

where *Y*_*it*_ is a dependent variable for person *i* at time* t*, *t* = 1,…, T_*i*_, where T_*i*_ is the number of observations for person *i*; a_*it*_ represents age of person *i* at time* t*; π_0*i*_ represents the level of a measure at age 10 (intercept) for person *i*; π_1*i*_ represents the instantaneous linear change at age 10 for person *i*; π_2*i*_ represents the acceleration in each growth trajectory for person *i*; e_*it*_ is the random within-person error for person *i* at time* t*. For linear growth models, π_2*i*_ is set as “0” and π_1*i*_ represents the linear growth rate for person *i* at time* t*. For Level-2 equations, the person-level covariates – age at Time 1, sex, and ID status – were added to predict the baseline (intercept) differences of measures, with age variables (linear and quadratic terms) of person *i* specified as random. Then, to evaluate effects of ID status on these trajectories, cross-level interaction terms between ID status and age were added to the models.

For each measure, we estimated four models, each building on the previous one. Model 1 estimated the linear age effect and also included the age of the autistic individual when the study began, sex, and co-occurring ID status. For Model 2, to assess whether age was best estimated as a linear or curvilinear function, we added a term of age-squared to the variables in Model 1. In Models 3 and 4, to assess whether the age-related linear or curvilinear trajectories differed by ID status, we additionally included interaction terms (age X ID in Model 3 and age-squared X ID in Model 4). Following the approach of Joiner, Bergeman, and Wang [51], the best model was selected based on log-likelihood tests for nested models (Model 1 versus Models 2, 3, and 4; Model 2 versus Model 4; Model 3 versus Model 4), and Akaike Information Criterion (AIC) and Bayesian Information Criterion (BIC) values for non-nested models (Models 2 versus Model 3). We report the results of the best model. Once the best model was selected, we tested the significance of random effects components – random intercept variance and random slope variance – using likelihood-ratio tests. Note that the random effects components of each best model showed that there were significant variations in initial levels of all outcomes among individuals with ASD even after the baseline age and the ID status were controlled. Similarly, there were significant between-individual differences in the age-related trajectories for all outcomes except for impairments in non-verbal communication, as we report below. Following these main analyses, we also conducted an exploratory analysis with each best fitting model to probe for potential cohort effects.

All analyses were conducted using Stata version 17.0 [52]. The level of significance was set at equal to or less than 0.05. The examination of distributions of the variables yielded no evidence of skewed distribution, with skewness ranging from -0.61 to 1.26. For the two count variables (the number of prescription psychometric and non-psychometric medications), additional analyses using random intercept Poisson regressions were also performed. Since the results were similar to those from the growth curve models treating the outcomes as continuous variables, we reported the results based on the continuous variable analyses.

We included all individuals in the study sample in the analysis, regardless of the number of data points they contributed. Those with just one data point contributed to the estimation of the intercepts and the effect of age at the start of the study on the intercept, but they did not contribute to estimates of the trajectories. With Maximum Likelihood (ML) estimation, the greater the number of data points, the more influential the case was to the estimation of the age-related trajectories. By fitting our models in a multilevel framework (as opposed to repeated measures ANOVA, for example), we were able to retain all observed data and accommodate missing observations under the same missing at random (MAR) assumptions as are adopted when using full information maximum likelihood (FIML) estimation in other modeling contexts. For descriptive purposes, the individual trajectories of each participant are portrayed in figures that were generated by the equation in the best model for each indicator of autism symptoms, behavioral functioning, and health.

## Results

### Descriptive findings

Table 3 presents descriptive data on the study sample, comparing autistic individuals who had co-occurring ID (*n* = 283) and those who had average or above-average intellectual functioning (*n* = 123) at baseline. Those who had ID were significantly older (by about five years) than those who did not have ID, but the two groups did not differ in sex. At the start of the study, there were significant differences between those who had co-occurring ID and those who had average or above-average intellectual functioning on most study variables. Those who had ID had significantly greater impairments in social reciprocity and communication (both verbal and non-verbal); were less independent in daily living skills; had greater levels of behavior problems; spent less social time with friends and neighbors; and were prescribed a greater number of non-psychotropic medications. The groups did not differ in repetitive behavior symptoms, ratings of health, or number of prescribed psychotropic medications.

**Table 3:** Characteristics of autistic participants by ID status^a^

Variable	Total (*n* = 406)	No ID group (*n* = 123)	ID group (*n* = 283)	t-value
Demographic Characteristics				
T1 Age	21.9 (9.4) [10, 52]	18.6 (7.7) [10, 47]	23.4 (9.7) [10, 52]	-4.87***
Sex (% males)	73.2%	73.2%	73.1%	Chi-sq = 0.00
Autism Symptoms				
ADI-R impairments in Social Reciprocity	15.6 (5.6) [2, 28]	12.2 (4.9) [2, 27]	17.1 (5.2) [5, 28]	-8.86***
ADI-R impairments in Non-Verbal Communication	5.01 (2.3) [0, 8]	4.11 (2.4) [0, 8]	5.40 (2.1) [0, 8]	-5.44***
ADI-R impairments in Verbal Communication	9.96 (3.5) [1, 20] (*n* = 299)	8.30 (3.3) [1, 16] (*n* = 122)	11.1 (3.1) [1, 20] (*n* = 177)	-7.48***
ADI-R Repetitive Behaviors	5.42 (2.4) [0, 12]	5.08 (2.5) [0, 11]	5.57 (2.4) [0, 12]	-1.86
Behavioral Functioning				
Activities of Daily Living (W-ADL)	19.6 (6.6) [3, 34]	24.1 (5.7) [10, 34]	17.7 (6.1) [3, 33]	9.81***
Maladaptive Behavior (SIB-R total score)	115.6 (11.2) [99, 153]	113.7 (10.9) [99, 150]	116.4 (11.3) [100, 153]	-2.17*
Time spent with friends/neighbors	1.28 (1.3) [0, 4]	1.71 (1.3) [0, 4]	1.10 (1.3) [0, 4]	4.10***
Health				
Health rating	3.19 (0.8) [1, 4]	3.12 (0.8) [1, 4]	3.21 (0.7) [1, 4]	-1.19
Number of Psychotropic Medications:	0.84 (0.9) [0, 6]	0.75 (0.9) [0, 3]	0.88 (1.0) [0, 6]	-1.03
Number of Non-psychotropic Medications	0.70 (0.9) [0, 4]	0.46 (0.8) [0, 3]	0.80 (1.0) [0, 4]	-3.57***

*ID* Intellectual Disability, *ADI-R* Autism Diagnostic Interview-Revised, *W-ADL* Waisman Activities of Daily Living Scale, *SIB-R* Scales of Independent Behaviors-Revised

^*^ *p* < .05; *** *p* < .001

^a^Means, standard deviation (in parentheses) and ranges (in brackets) are reported unless the variable is marked with (%)

### Trajectories of age-related change

#### Age-related trajectories in autism symptoms

##### ADI-R impairments in social reciprocity

Model 3 was the best fitting model in the prediction of age-related trajectories of impairments in social reciprocity (see Table 4A).

**Table 4:** Best fitting growth curve models for autism symptom measures

	4A. ADI-R Social Reciprocity Impairment	4B. ADI-R Communication (non-Verbal) Impairment	4C. ADI-R Communication (Verbal) Impairment (*n* = 318)	4D. ADI-R Repetitive Behavior
Best fitting Model	Model 3	Model 1	Model 3	Model 4
Fixed Effects				
T1 age	.11 (.04)**	.04 (.01)**	.11 (.03)***	.08 (.02)***
Sex (Female = 1)	-.36 (.55)	-.32 (.23)	-.13 (.40)	-.02 (.23)
ID (ID = 1)	3.42 (.70)***	1.14 (.22)***	2.20 (.53)***	.01 (.42)
Age	-.17 (.04)***	-.001 (.010)	-.15 (.03)***	-.27 (.05)***
Age-squared	–	–	–	.004 (.001)**
Age x ID	.18 (.04)***	–	.09 (.03)**	.17 (.05)**
Age-sq x ID	–	–	–	-.004 (.001)***
Constant	12.5 (.60)***	3.78 (.21)***	8.51 (.41)***	5.81 (.34)***
Random Effects^a^				
Var. (Age)	.037 [.020, .067]	–	.016 [.007, .036]	.003 [.001, .015]
Var. (intercept)	20.6 [15.8, 27.1]	3.48 [2.98, 4.08]	9.09 [6.50, 12.7]	3.98 [2.85, 5.57]
Cov. (Age, intercept)	-.280 [-.576, .015]	–	-.154 [-.324, .016]	-.053 [-.129, .023]
Var. (Level-1 residual)	7.39 [6.78, 8.05]	1.73 [1.60, 1.87]	4.22 [3.82, 4.67]	2.70 [2.48, 2.93]

*ID* Intellectual Disability, *ADI-R* Autism Diagnostic Interview-Revised

^**^ *p* < 0.01; *** *p* < .001

^a^Estimated variances (Var.) and covariances (Cov.) of random parts of the mixed models are reported with 95% confidence intervals in brackets

**Fig. 1:**
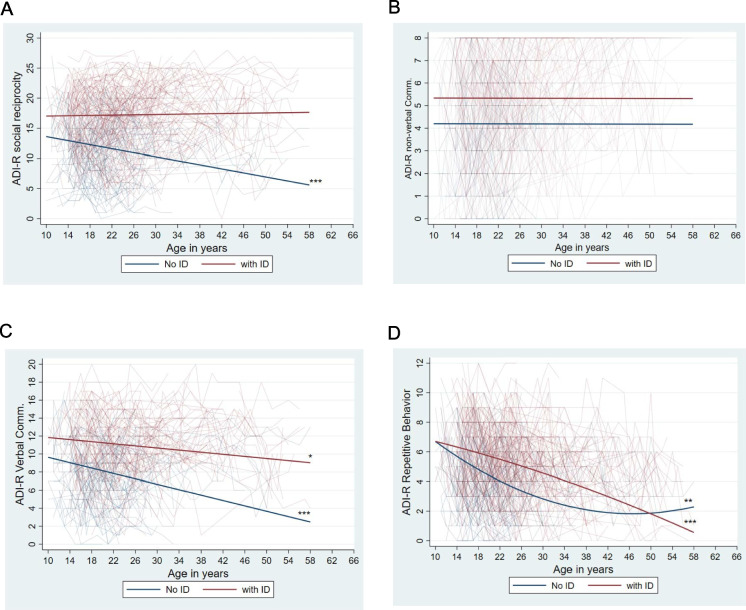
Individual and average trajectories of change in autism symptom measures by ID status. **A** ADI-R Social reciprocity impairment. **B** ADI-R communication (non-Verbal) impairment. **C** ADI-R communication (Verbal) impairment (*n* = 299). **D** ADI-R repetitive behavior impairment. * *p* < .05; ** *p* < .01; *** *p* < .001. note: *ID* Intellectual Disability, *ADI-R* Autism Diagnostic Interview-Revised

The associations of age and impairments in social reciprocity were linear and differed for those with and without ID (age X ID coefficient = 0.18, *p* < 0.001). As descriptively illustrated in Fig. 1A, those who did not have ID showed decreasing impairments in social reciprocity, whereas for those who had ID the level of impairments did not change from adolescence into adulthood and midlife.

##### ADI-R impairments in non-verbal communication

Model 1 was the best fitting model for impairments in non-verbal communication (see Table 4B). There was no significant age-related change in the level of impairment in non-verbal communication, nor were there differences by ID status (Fig. 1B). The random effects components of the growth curve model showed that, while there were significant differences between individuals with ASD for the initial level of impairment in non-verbal communication, there were no inter-individual differences in the age-related trajectory, which was virtually flat.

##### ADI-R impairments in verbal communication

Similar to the patterns observed for impairments in social reciprocity, Model 3 was the best fitting model for impairments in verbal communication (see Table 4C). The age-related trajectories of impairments in verbal communication were linear, with different slopes for those who had ID and those who did not (age X ID coefficient = 0.09, *p* < 0.01). Based on visual description of Fig. 1C, these impairments were more substantial for those with ID at all ages. Importantly, although impairments in verbal communication significantly decreased for both groups from their teenage years through midlife and beyond, for those who did not have ID the rate of decrease in impairments was much greater than for those who had ID.

##### ADI-R repetitive behavior

Model 4 was the best fitting model in the prediction of age-related trajectories of repetitive behaviors (see Table 4D). Autistic individuals with and without ID differed in the slopes of their trajectories, as indicated by the age-squared X ID coefficient (-0.004, *p* < 0.001). As described in Fig. 1D, for individuals with ID, on average there was a linear age-related decrease in repetitive behaviors. In contrast, for those who did not have ID, the association between repetitive behaviors and age was curvilinear. The figure illustrates that, among those who did not have ID, repetitive behaviors decreased in severity during adolescence and the early years of adulthood, and increased in midlife and beyond. Additionally, visual inspection of the figure suggests that although the severity of repetitive behaviors was greater among those with ID for most of the adult years, later in the life course the symptoms of those who did not have ID ultimately exceeded the level of symptoms for those with ID.

#### Age-related trajectories of behavioral functioning

##### Activities of daily living

Model 4 was the best fitting model in the prediction of age-related trajectories of activities of daily living (see Table 5A).

**Table 5:** Best fitting growth curve models for behavioral functioning measures

	5A. Activities of Daily Living (W-ADL)	5B. Maladaptive Behavior (SIB-R total score)	5C. Time Spent with Friends/Neighbors
Best fitting Model	Model 4	Model 2	Model 2
Fixed Effects			
T1 age	.08 (.04)*	-.04 (.05)	.01 (.01)
Sex (Female = 1)	-.62 (.62)	1.74 (.88)*	.26 (.12)*
ID (ID = 1)	-4.67 (.84)***	5.98 (.88)***	-.64 (.12)***
Age	.58 (.06)***	-.67 (.07)***	.03 (.01)**
Age-squared	-.011 (.001)***	.009 (.002)***	-.001 (.000)*
Age x ID	-.45 (.07)***	–	–
Age-sq x ID	.007 (.002)**	–	–
Constant	20.7 (.71)***	116.0 (1.1)***	1.40 (.13)***
Random Effects^a^			
Var. (Age)	.034 [.024, .048]	.161 [.120, .216]	.002 [.001, .003]
Var. (intercept)	26.0 [20.7, 32.7]	114.4 [93.2, 140.5]	1.14 [.832, 1.56]
Cov. (Age, intercept)	-.259 [-.475, -.043]	-3.38 [-4.34, -2.42]	-.024 [-.040, -.009]
Var. (Level-1 residual)	6.17 [5.66, 6.74]	33.6 [31.4, 36.0]	.979 [.912, 1.05]

*ID* Intellectual Disability, *W-ADL* Waisman Activities of Daily Living Scale, *SIB-R* Scales of Independent Behaviors-Revised

^*^ *p* < .05; ** *p* < .01; *** *p* < .001

^a^Estimated variances (Var.) and covariances (Cov.) of random parts of the mixed models are reported with 95% confidence intervals in brackets

**Fig. 2:**
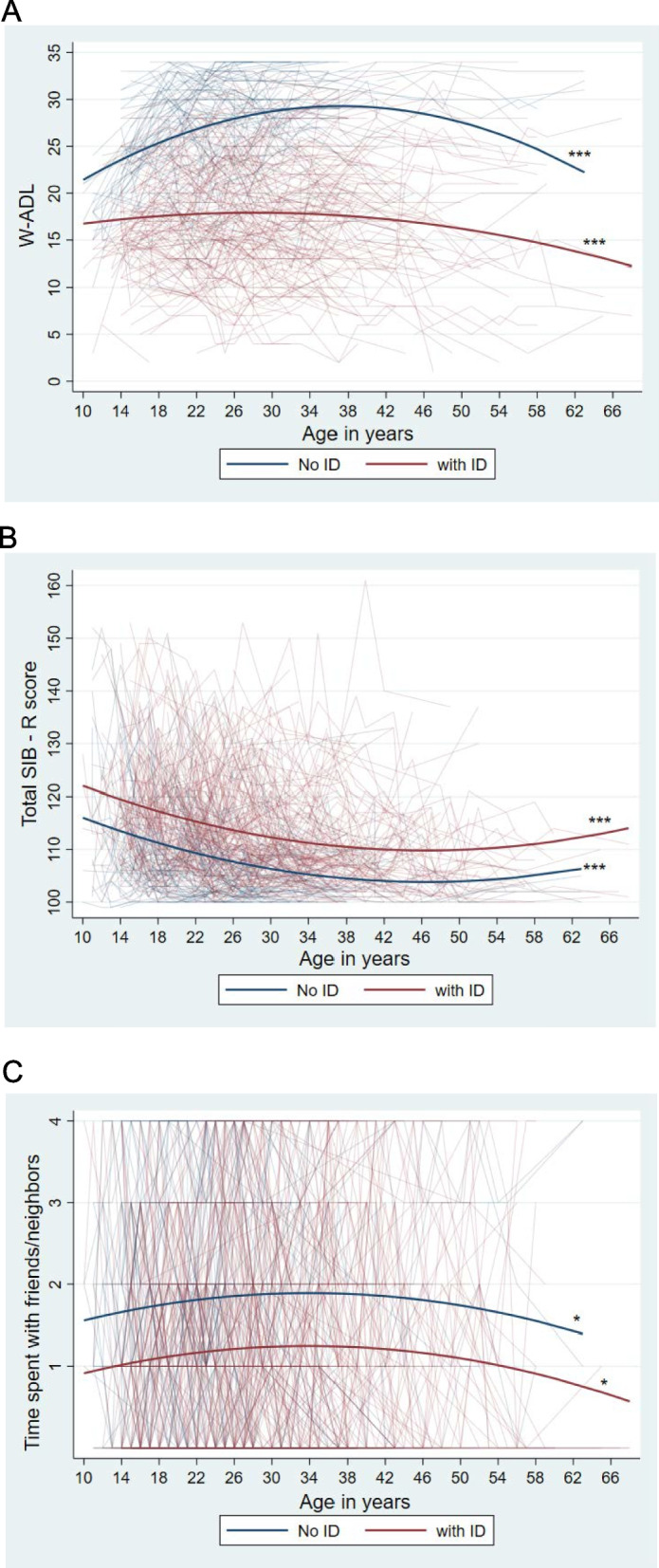
Individual and average trajectories of change in behavioral functioning measures by ID status. **A** Activities of daily living (W-ADL). **B** Maladaptive behavior (SIB-R total score). **C** Time spent with friends/neighbors. * *p* < .05; *** *p* < .001. note: *ID* Intellectual Disability, *W-ADL* Waisman Activities of Daily Living Scale, *SIB-R* Scales of Independent Behaviors-Revised

The age-related trajectories in ADL independence for both those with ID and those who did not have ID were curvilinear. For both ID groups, on average, ADL independence increased during the adolescent and early adult years, but decreased in midlife and beyond. However, the slopes of the two groups differed, as indicated by the significant age-squared X ID coefficient (0.007, *p* < 0.01). Visual description of Fig. 2A indicated a steeper increase in ADL skills during adolescence and a more marked decrease during midlife and beyond for those who did not have ID than for those with ID.

##### Maladaptive behavior

Model 2 was the best fitting model in the prediction of age-related trajectories of maladaptive behavior (see Table 5B). A curvilinear age-related trajectory was indicated by the significant age-squared coefficient (0.009, *p* < 0.001), and the slope of the trajectory did not differ for those with ID and those who did not have ID. Visual inspection of Fig. 2B suggests that, for both ID groups, the severity of maladaptive behavior decreased during the adolescent and early adult years, but increased during midlife and beyond.

##### Social participation

Model 2 was the best fitting model in the prediction of age-related trajectories of socializing with friends and neighbors (see Table 5C). The trajectories for both those with ID and those who did not have ID were curvilinear, as indicated by the significant age-squared coefficient (-0.001, *p* < 0.05). For both ID groups, the frequency of socializing with friends and neighbors increased during the adolescent and early adult years and decreased in midlife and beyond (see Fig. 2C). Descriptively, at around age 40, the frequency of spending time with friends and neighbors was approximately once or twice a month for those who did not have ID, but only around several times a year for those who had ID.

#### Age-related trajectories of health

##### Health ratings

Model 1 was the best fitting model in the prediction of the age-related trajectory in health ratings (see Table 6A).

**Table 6:** Best fitting growth curve models for health measures

	6A. Health Rating	6B. Psychotropic Medications	6C. Non-psychotropic Medications
Best fitting Model	Model 1	Model 3	Model 4
Fixed Effects			
T1 age	.01 (.00)**	-.04 (.01)***	-.07 (.01)***
Sex (Female = 1)	-.08 (.06)	-.10 (.12)	.22 (.11)
ID (ID = 1)	.06 (.06)	.01 (.15)	-.15 (.18)
Age	-.02 (.00)***	.03 (.01)***	-.02 (.02)
Age-squared	–	–	.002 (.000)***
Age x ID	–	.03 (.01)**	.09 (.02)***
Age-sq x ID	–	–	-.0015 (.0005)**
Constant	3.25 (.06)***	.90 (.13)***	.76 (.15)***
Random Effects^a^			
Var. (Age)	.000 [.000, .001]	.003 [.003, .004]	.003 [.003, .005]
Var. (intercept)	.284 [.211, .381]	1.09 [.869, 1.37]	.672 [.462, .977]
Cov. (Age, intercept)	-.005 [-.009, -.001]	-.030 [-.042, -.017]	-.020 [-.034, -.007]
Var. (Level-1 residual)	.246 [.230, .263]	.406 [.379, .435]	.771 [.720, .825]

*ID* Intellectual Disability

^**^ *p* < .01; *** *p* < .001

^a^Estimated variances (Var.) and covariances (Cov.) of random parts of the mixed models are reported with 95% confidence intervals in brackets

**Fig. 3:**
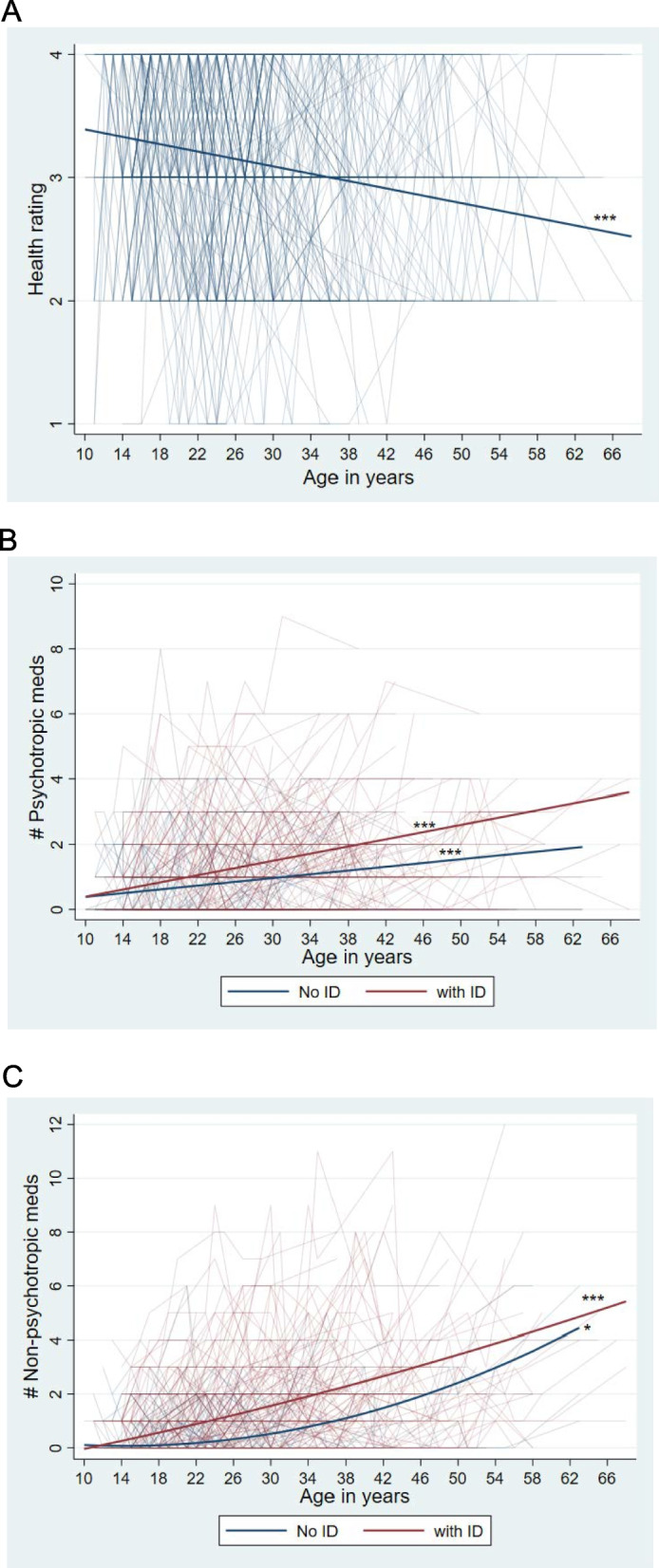
Individual and average trajectories of change in health measures by ID Status. **A** Health rating. **B** The number of psychotropic medications. **C** The number of non-psychotropic medications. * *p* < .05; *** *p* < .001. note: *ID* Intellectual Disability

Unlike most other indicators, the age-related trajectories did not differ between those who had co-occurring ID and those who did not have ID, either in level or slope. The association between age and health ratings was linear and negative (-0.02, *p* < 0.001), as illustrated in Fig. 3A. Descriptively, during adolescence, autistic individuals averaged between good and excellent health, whereas by the late 30 s and thereafter ratings averaged between fair and good health. Few were rated as having poor health at any point of the study.

##### Number of psychotropic medications

Model 3 was the best fitting model in the prediction of the age-related trajectory of psychotropic medications that were prescribed for mental health symptoms (Table 6B). For both those who had ID and those who did not, the age-related trajectories of the number of psychotropic medications increased linearly, but the rate of increase was greater for those who had ID, as indicated by the significant of the age x ID interaction coefficient (0.03, *p* < 0.01). Visual inspection of Fig. 3B suggests that during their teens, the two groups did not differ in the number of psychotropic medications they were prescribed, but by late midlife individuals who had ID averaged approximately three psychotropic medications whereas those who did not have ID averaged nearly two.

##### Number of non-psychotropic medications

Model 4 was the best fitting model in the prediction of the age-related trajectory of non-psychotropic medications that were prescribed for physical health symptoms (see Table 6C). Autistic individuals with and without ID differed in the slopes of their trajectories, as indicated by the age-squared X ID interaction coefficient in Model 4 (-0.0015, *p* < 0.01). As descriptively illustrated in Fig. 3C, for individuals with ID, on average there was a linear age-related increase in the number of non-psychotropic medications they were prescribed. In contrast, for those who did not have ID, the association between age and the number of non-psychotropic medications was curvilinear, increasing more rapidly starting around midlife. Although during their early teens, the two groups did not differ in the number of non-psychotropic medications they were prescribed, by late midlife, those who had ID were prescribed an average of approximately four non-psychotropic medications, whereas those who did not have ID were prescribed an average of approximately three.

### Exploration of cohort differences in age-related trajectories

As an exploratory follow-up analysis, we evaluated whether the age-related trajectories estimated in the best fitting models differed depending on the age of the autistic person when the study began. The interaction effect between Time 1 age and the age trajectory term (either age main effect or age X ID interaction effect) was tested in the best model for each dependent variable (see Supplemental Materials for the results). A significant interaction would suggest differences in trajectories between those who were younger versus older when the study began.

The only significant interaction effect was for independence in ADL skills (Time 1 age X age-squared X ID status = -0.0004, *p* < 0.05). The coefficient suggests that for those who were adolescents at Time 1, independence was increasing during the study period, whereas those who entered the study in midlife had already peaked in their ADL independence, especially among those who did not have ID.

## Discussion

This study described age-related trajectories using an accelerated longitudinal design that estimated trends over a 60-year period. It drew from data collected prospectively over 22 years on a large community-based sample of autistic individuals and captured the heterogeneity of diagnosed autism at the time the study began. The analysis provided preliminary insights about patterns of change through adulthood, midlife, and into the early years of older age.

We investigated the extent of age-related change during the decades between adolescence and midlife and beyond, whether there were improvements or worsening in these domains, whether the change was linear or curvilinear, and how those with and without an ID diagnosis differed. To summarize the findings, there were significant trajectories of age-related change for all measures for both those with and without ID except impairments in non-verbal communication and impairments in social reciprocity; for these measures, there was no change for individuals who had ID. This dominant pattern of age-related change underscores the importance of longitudinal research over the full adult period for autistic individuals, a stage of life that has been beyond the upper age limit of most prior studies of autism. This is one of the notable contributions of the present research – that adulthood is not a static period for autistic individuals, although the patterns reported here need replication and extension further into old age.

However, this is not an uncomplicated story. In this two-decade prospective study, three patterns of change were observed, reflecting both the direction and slope of change. The first pattern was *significant improvement* over the adolescent and adult years, evident for two of the measures that comprise the diagnostic algorithm of autism (impairments in social reciprocity for those who do not have ID, and impairments in verbal communication regardless of ID status), both of which became linearly less severe as individuals aged. This pattern of reduction in autism symptom severity associated with advancing age is consistent with much past research (e.g., [18, 53]), although there is some evidence that symptoms may increase in certain subgroups between early and middle childhood [54]. By extending the longitudinal pattern into the midlife period and beyond, the present study confirms that trajectories established earlier in the life course can be extended out to accurately reflect change during these later periods. This confirmation of earlier trajectories can only be reached in retrospect, and future research is needed to determine whether the linear reduction in symptoms continues into old age.

Second, a prominent pattern of *significant worsening* over the adolescent and adult years was observed for all three indicators of health – ratings of health worsened and the numbers of prescribed medications for both mental health and physical health symptoms increased with advancing age. Although these patterns are characteristic of the general population and although past cross-sectional research has documented the poorer health of autistic individuals than their age peers [55, 56], whether worsening health begins earlier for those diagnosed with autism as compared with the general population cannot be determined from this study. This is a critical area for future research, especially since there have been population-level studies that suggest a shorter lifespan for individuals diagnosed with autism [24, 26].

These first two patterns (improvement, worsening) reflect continuity across the study period, when patterns that are evident in adolescence and early adulthood continue into midlife and older age. In contrast, for the other measures we evaluated, there was a pattern of discontinuity during the study period, reflecting *improvement during adolescence into adulthood, followed by a levelling off, and then worsening in midlife and beyond*. This pattern of discontinuity was characteristic of activities of daily living, maladaptive behaviors, repetitive behavior symptoms, and socializing with friends and relatives. At midlife, independence in daily living skills peaked, behavior problems and repetitive behavior were at their lowest level, and interaction with friends and neighbors most frequent. These indicators of improved functioning in early adulthood have been reported by other studies of autism [57, 58]. However, there have been few if any prior longitudinal reports of increased difficulties after their mid-thirties to mid-forties, trending back toward levels manifested during adolescence. These non-linear patterns of change reflect the observation made by Lachman et al. [32] regarding midlife as a pivotal time, linking periods of growth and decline. Furthermore, this pattern of gain and loss underscores the importance of taking the long-view on questions of adulthood for autistic individuals, as patterns of gain that are evident during early adulthood may not be sustained after midlife. Even when there is continuity, the slope of change may signal increasing difficulties associated with advancing age, as was observed for medications prescribed for physical health symptoms for autistic adults who did not have ID. Studies that base inferences on short periods of the adult years have the potential of misspecification of the long-term trajectory and direction of change, and may also underestimate the support needs of older autistic adults.

There are important implications of the present study for clinical practice, suggesting how support needs of autistic adults likely change with advancing age. For example, the observed gains in daily living skills and time socializing with friends, along with reductions in maladaptive behaviors, suggest that the years before midlife could be considered a time of thriving for many autistic adults; there may be a relatively good match of services to needs, on average, during this period of the life course. However, the observed declines in functioning at midlife and beyond may signal a need for a higher level of, or different constellation of, services for autistic adults during this period. Service plans that were originally designed and implemented during the transition into adulthood likely need to be reassessed and adapted to keep pace with the changing needs of autistic individuals and their families as they move into old age. Examining the interplay of formal services, natural supports, and outcomes for aging adults, along with the efficacy of targeted interventions for this period of the life course, will be critical areas of future research.

An additional important clinical implication of this research is to make it possible for autistic adults, their families, and society to plan for the future. The population diagnosed as having autism has increased exponentially since around the year 1990 [59], and the members of this “diagnostic boom” generation are now approaching or are well-into midlife. Developmental trajectories over the life course have the potential of revealing turning points, such as exiting high school, moving away from the parental home, or loss of a parent, when vulnerabilities in key domains may increase or decrease, signaling differential need for services and supports [60, 61].

The results of the present study provide confirmatory evidence supporting much past research indicating that autistic adults who have ID have significantly poorer functioning than those without ID. Our results provide novel data indicating that the *rate of age-related change* differed for the two groups (i.e., a different slope). Those who did not have ID had sharper increases in independence in activities of daily living during adolescence and early adulthood and then more rapid decreases than those who had ID. Additionally, for those who had average or above average intellectual functioning, after midlife their repetitive behaviors increased with advancing age and became worse than those who had co-occurring ID. The two ID groups differed also in the rate of increase in the number of medications they were prescribed; surprisingly, those who did *not* have ID had a more rapid increase in medications prescribed for physical health problems after midlife. Stepping back from the details, these patterns suggest that those who have ID have a very different adult life course than those who have average or above-average intellectual functioning. Notably, in the present sample, although those with ID had greater impairments, for those who had average or above average intellectual functioning, worsening in some measures accelerated in their later years.

What are the implications of these divergent patterns of the adult life course? At the most basic level, those who have ID, as well as those who are minimally verbal, generally require a different constellation of supports and services throughout adulthood than those who can live independently, and this may also be true for their families, who are key to the provision of support across the life course. However, there are signs that autistic adults with average or above average intellectual functioning may have accelerating difficulties in their aging years, and thus their needs in specific areas may also increase as they age. Thus, it behooves service providers, policy makers, and researchers not to focus on only one group within the overall population of autistic individuals, as their differences in key aspects of the life course are consequential. Although the present study includes more of those who have ID than those who do not, our examination of differences between these groups revealed where in specific the groups followed divergent life course patterns (e.g., impairments in social reciprocity) and where they are similar (e.g., overall health ratings). Given these different life course patterns and needs, generalizing from those who do not have ID to those who do is not warranted, and has the potential to lead to conclusions or recommendations that do not reflect the needs of these individuals. The reverse pattern of generalization is also not appropriate.

The present findings also underscore the value of examining multiple measures longitudinally, rather than inferring change from a few dependent variables, as we found that the pattern of change across the life course tends to differ for different variables. It has been well-established in the field of autism that the population with this diagnosis is heterogeneous, even from the earliest years of childhood [62]. Similarly, it has been well-established in the field of gerontology that populations become more heterogeneous as they age due to the cumulative impact of both intrinsic and extrinsic factors [63]. This heterogeneity includes diversity among individuals in the rate of age-related change as well as diversity of patterns of change across indicators of aging. A recent large-scale longitudinal study of aging in the general population, based on the 30,000-member Canadian Longitudinal Study of Aging, highlighted that although overall heterogeneity increases with age, it does not do so uniformly across all domains [64], similar to the findings of the present study. Future research is needed to elucidate the factors that can account for heterogeneity in patterns of aging among autistic individuals.

Cohort effects could explain this difference in heterogeneity in trajectories, although in the present study, there was little evidence of cohort effects on the trajectories other than the significant Time 1 age effects that indicated that those who were younger at the start of the study had fewer impairments. This is not to say that cohort effects do not exist for individuals diagnosed with autism, but rather that they were not evident with respect to these age-related trajectories during the time period of the present study. Notably, all of the autistic individuals in this study were diagnosed before the change in the autism definition in the *Diagnostic and Statistical Manual of Mental Disorders*, fifth edition (DSM-5), which has led to substantial cohort differences, and thus generalization of our study findings to subsequent generations should be cautious.

Future research could approach the study of age-related change in autistic adults via the inclusion of trajectories in one domain as covariates or predictors of trajectories of other domains. For example, covarying trajectories of “autism” related symptoms (e.g., impairments in social reciprocity) in the estimation of trajectories of other characteristics (e.g., activities of daily living) could possibly yield a more nuanced understanding of how these might co-occur or how one might explain or account for the other. However, caution is needed prior to implementing such an approach, as the shape of the age-related trajectories of different outcomes are not identical. For example, the shape of the trajectory of autism symptoms in the present analysis, differed from the shape of the trajectory of activities of daily living, with the former being continuous age-related improvement and the latter being improvement until midlife followed by worsening. Thus, covarying characteristics that have different trajectories than outcomes might skew understanding of how these characteristics change over the adult years.

### Limitations and strengths

This study is not without its methodological limitations. Like other long-term longitudinal studies, attrition is an important limitation, although we retained nearly half of the sample over the full two-decade study. Notably, the accelerated longitudinal design approach to statistical analysis made it possible to include the entire sample of 406 autistic individuals, although the length of each person’s trajectory was affected by the duration of their retention in the study. Some long-term longitudinal studies of aging such as the Midlife in the United States (MIDUS) study (www.midus.wisc.edu) have incorporated “refresher” cohorts to replace attrition cases and to evaluate cohort effects directly. This strategy would be valuable in studies of midlife and aging in autism.

Generalization of the present research results must be tempered by several factors. One is the lower representation of the full spectrum of autism as currently defined in the DSM-5. In addition, there were fewer data points reflecting autistic individuals in midlife and the early years of older age than adolescence through midlife. Although the data points reflecting early old age are sparser than earlier in the life course, these data constitute the unique contribution of the present study. Nevertheless, the age-related patterns reported here warrant replication. The lack of ethnic and racial diversity is an additional significant limitation, although the diversity of participants in socioeconomic status, particularly the inclusion of families living below the poverty line and the number of families where the mother does not have a college degree, is an important other source of diversity.

The type of data included in this report relied on parent (mother) reports and warrants replication with other data sources. Some measures selected for the present analysis were designed to be rated by others (e.g., ADI-R), while other measures were objective (e.g., number of prescription medications). Nevertheless, collecting data from other reporters, including directly from autistic individuals whenever possible, is an important next step in this line of research, as is the collection of biological indicators of aging.

Juxtaposed against these limitations are several strengths of the present study, including its long longitudinal study period extending over two decades, the prospective repeated measures approach, the inclusion of a community-based sample that was heterogeneous in age, ID status, and socioeconomic status, and the focus that spanned adolescence and adulthood and extended well into midlife and beyond. These limitations and strengths point the way toward future research.

### Future research directions

Among the most important directions for future research are the need for replication and extension of the data collection period further into old age. Deeper investigation of the differences in the life course of those with and without ID, those who are minimally verbal, and those with other constellations of challenges is also needed to understand the distinct biopsychosocial characteristics of autism subgroups and their divergent needs as they age. Relatedly, future research on ethnically and racially diverse samples should be a priority.

Benchmarking with general population data is needed to determine if patterns observed here differ from the general population. Review of published data is a possible first step. For example, as the measure of time spent with friends and neighbors in the present sample was taken from a nationally-representative sample of the general population (the National Survey of Families and Households, www.ssc.wisc.edu/nsfh/), we can compare the frequency in the present sample to published national patterns (i.e., once or twice per month for the autistic adults in the present sample at around age 40 compared to five times per month at a mean age of 42 for the national sample, respectively [65]). Future studies with well-matched comparison groups would represent an important next step in benchmarking, although few studies of autism in adulthood employ this important research design approach (an exception is the Australian Longitudinal Study of Autistic Adults [66]).

The present study demonstrates notable age-related changes in the trajectories of outcomes. However, the random effect components of growth curve models revealed considerable variability between individuals in the initial levels and rates of change for all outcomes. Notably, ID status accounted for some of this variability, as evidenced by significant interactions between ID status and age (or the age quadratic term) for autism symptoms (impairments in social reciprocity, verbal communication, and repetitive behaviors), daily living skills, and number of non-psychotropic medications. Future research endeavors should aim to explore additional individual characteristics and contextual factors that could account for such interindividual variability.

Additionally, investigating the associations between the trajectories described here with adult outcomes (e.g., employment/retirement, residential independence) is an important line of future research. Latent class approaches may be helpful in this line of inquiry. Extending the accelerated longitudinal approach to other measures that potentially reflect change as autistic individuals age (e.g., cognitive, biomarkers, brain imaging indicators of aging) will be potentially important for future planning. Evaluating how the trajectories predict longevity and mortality would be a critical direction in life course research on autism. In future research, it will be valuable to explore associations *among* the trajectories to discover whether changes in one trajectory are correlated with changes in another, and furthermore whether some prior trajectories predict subsequent changes. Although this is beyond the scope of the present study, this is an important next step.

Lastly, it will be important to further conceptualize the definition of midlife for autistic people. As noted, it is referred to in general literature as a period that is roughly defined as spanning the decades between 40 and 60 *plus or minus 10 years*. As autistic adults may have poorer health and a shorter lifespan than their age peers, perhaps the beginning of midlife should be shifted downward for them. The slopes in the figures presented in this study suggest this possibility.

## Conclusion

The present investigation is a preliminary step in the study of midlife and aging in those diagnosed with autism. Both the complexity and heterogeneity of autism during the period of the life course extending from adolescence into midlife and the earliest years of old age are highlighted by the present findings. Although aging in autism is a new research focus, this subgroup of the population is not new; it has always existed. Now is the time to prioritize research on this life stage, giving it the same careful attention that has revealed developmental processes during early childhood.

### Supplementary Information

**Additional file 1: Supplemental Materials. **Mixed growth curve models with mother respondents only, and testing cohort effects.

## Data Availability

The dataset analyzed in this study is not publicly available per IRB. When they enrolled in the study and at all subsequent rounds of data collection, participants were assured that raw data would not be shared, due to the sensitive nature of the study.

## Abbreviations

ID: Intellectual disability
ADI-R: Autism Diagnostic Interview-Revised
PDD-NOS: Pervasive Developmental Disorder-Not Otherwise Specified
ADL: Activities of Daily Living
W-ADL: Waisman Activities of Daily Living Scale
SIB-R: Scales of Independent Behaviors-Revised
CNS: Central Nervous System
GI: Gastrointestinal
WRIT: Wide Range Intelligence Test
ALD: Accelerated Longitudinal Design
AIC: Akaike Information Criterion
BIC: Bayesian Information Criterion
DSM-5: Diagnostic and Statistical Manual of Mental Disorders, fifth edition
MIDUS: Midlife in the United States
