# Supplementary material for: Autism through midlife: trajectories of symptoms, behavioral functioning, and health
Source: J Neurodev Disord. 2023 Nov 3;15:36. doi: 10.1186/s11689-023-09505-w (PMC10623813; doi:10.1186/s11689-023-09505-w)
Supplement: Supplementary file 1 — Additional file 1: Supplemental Materials. Mixed growth curve models with mother respondents only, and testing cohort effects. [file 11689_2023_9505_MOESM1_ESM.docx]

[Supplemental Material #1]

The tables present results of mixed growth curve models based only on data collected from mothers.

Table S1.1. Best Fitting Growth Curve Models for Autism Symptom Measures: Mother Respondents Only.

|  | S1.1A. ADI-R Social Reciprocity Impairment | S1.1B. ADI-R Communication (non-Verbal) Impairment | S1.1C. ADI-R Communication (Verbal) Impairment | S1.1D. ADI-R Repetitive Behavior Impairment |
| --- | --- | --- | --- | --- |
| Best fitting Model | Model 3 | Model 1 | Model 3 | Model 4 |
| Fixed Effects |  |  |  |  |
| T1 age | .11 (.04)** | .05 (.01)** | .12 (.03)*** | .08 (.02)*** |
| Sex (Female=1) | -.35 (.55) | -.33 (.23) | -.12 (.40) | -.01 (.23) |
| ID (ID = 1) | 3.44 (.71)*** | 1.13 (.23)*** | 2.21 (.53)*** | .01 (.42) |
| Age | -.17 (.04)*** | -.003 (.010) | -.15 (.03)*** | -.27 (.04)*** |
| Age-squared | -- | -- | -- | .004 (.001)** |
| Age x ID | .18 (.04)*** | -- | .08 (.03)** | .17 (.05)** |
| Age-sq x ID | -- | -- | -- | -.004 (.001)*** |
| Constant | 12.5 (.60)*** | 3.79 (.21)*** | 8.45 (.42)*** | 5.83 (.34)*** |
| Random Effects |  |  |  |  |
| Var. (Age) | .036 [.020, .067] | -- | .014 [.006, .033] | .002 [.001, .017] |
| Var. (intercept) | 21.1 [16.1, 27.7] | 3.59 [3.06, 4.21] | 9.27 [6.63, 12.9] | 4.07 [2.91, 5.69] |
| Cov. (Age, intercept) | -.287 [-.589, .014] | -- | -.140 [-.312, .032] | -.049 [-.124, .026] |
| Var. (Level-1 residual) | 7.20 [6.60, 7.86] | 1.65 [1.53, 1.78] | 4.09 [3.70, 4.53] | 2.68 [2.46, 2.91] |

** p < 0.01; *** p < 0.001

a. Estimated variances (Var.) and covariances (Cov.) of random parts of the mixed models are reported with 95% confidence intervals in brackets.

note: *ID* Intellectual Disability, *ADI-R* Autism Diagnostic Interview-Revised.

Table S1.2. Best Fitting Growth Curve Models for Behavioral Functioning Measures: Mother Respondents Only.

|  | S1.2A. Activities of Daily Living (W-ADL) | S1.2B. Maladaptive Behavior (SIB-R total score) | S1.2C. Time Spent with Friends/Neighbors |
| --- | --- | --- | --- |
| Best fitting Model | Model 4 | Model 2 | Model 2 |
| Fixed Effects |  |  |  |
| T1 age | .08 (.04)* | -.04 (.05) | .01 (.01) |
| Sex (Female=1) | -.63 (.63) | 1.61 (.89) | .27 (.12)* |
| ID (ID = 1) | -4.70 (.85)*** | 6.12 (.89)*** | -.66 (.12)*** |
| Age | .58 (.06)*** | -.67 (.07)*** | .03 (.01)** |
| Age-squared | -.011 (.002)*** | .009 (.002)*** | -.001 (.000)* |
| Age x ID | -.45 (.07)*** | -- | -- |
| Age-sq x ID | .007 (.002)** | -- | -- |
| Constant | 20.7 (.71)*** | 116.1 (1.1)*** | 1.39 (.13)*** |
| Random Effects |  |  |  |
| Var. (Age) | .035 [.025, .050] | .158 [.117, .214] | .002 [.001, .003] |
| Var. (intercept) | 26.4 [20.9, 33.4] | 116.4 [94.6, 143.4] | 1.14 [.821, 1.58] |
| Cov. (Age, intercept) | -.266 [-.490, -.042] | -3.40 [-4.38, -2.43] | -.025 [-.041, -.009] |
| Var. (Level-1 residual) | 6.19 [5.66, 6.76] | 34.1 [31.8, 36.5] | .986 [.917, 1.06] |

* p < .05; ** p < 0.01; *** p < 0.001

a. Estimated variances (Var.) and covariances (Cov.) of random parts of the mixed models are reported with 95% confidence intervals in brackets.

note: *ID* Intellectual Disability, *W-ADL* Waisman Activities of Daily Living Scale, *SIB-R* Scales of Independent Behaviors-Revised.

Table S1.3. Best Fitting Growth Curve Models for Health Measures: Mother Respondents Only.

|  | S1.3A. Health Rating | S1.3B. # Medications: Psychotropic | S1.3C. # Medications: Non-psychotropic |
| --- | --- | --- | --- |
| Best fitting Model | Model 1 | Model 3 | Model 4 |
| Fixed Effects |  |  |  |
| T1 age | .01 (.00)** | -.05 (.01)*** | -.07 (.01)*** |
| Sex (Female=1) | -.08 (.06) | -.10 (.12) | .20 (.11) |
| ID (ID = 1) | .05 (.06) | .01 (.15) | -.11 (.18) |
| Age | -.02 (.00)*** | .03 (.01)*** | -.02 (.02) |
| Age-squared | -- | -- | .002 (.000)*** |
| Age x ID | -- | .03 (.01)** | .09 (.02)*** |
| Age-sq x ID | -- | -- | -.0014 (.0005)** |
| Constant | 3.25 (.06)*** | .90 (.13)*** | .76 (.15)*** |
| Random Effects |  |  |  |
| Var. (Age) | .000 [.000, .001] | .003 [.003, .004] | .003 [.003, .005] |
| Var. (intercept) | .297 [.222, .397] | 1.08 [.855, 1.36] | .660 [.447, .974] |
| Cov. (Age, intercept) | -.005 [-.009, -.001] | -.030 [-.042, -.017] | -.020 [-.033, -.005] |
| Var. (Level-1 residual) | .243 [.227, .260] | .414 [.386, .444] | .785 [.733, .841] |

** p < 0.01; *** p < 0.001

a. Estimated variances (Var.) and covariances (Cov.) of random parts of the mixed models are reported with 95% confidence intervals in brackets.

note: *ID* Intellectual Disability.

[Supplemental Material #2]

The tables present results of mixed growth curve models testing if the age trajectories estimated in the best fitting model in Tables 3, 4, and 5 in the main text differ by Time 1 age. To test this, the interaction term of Time 1 age with the age trajectory term (age main effect term or age X ID interaction effect term) from the best fitting model was added to the models. The coefficients of this interaction term are marked with bold face in the tables below.

Table S2.1. Effects of Time 1 Age on the Best Fitting Growth Curve Models for Autism Symptom Measures.

|  | S2.1A. ADI-R Social Reciprocity Impairment | S2.1B. ADI-R Communication (non-Verbal) Impairment | S2.1C. ADI-R Communication (Verbal) Impairment | S2.1D. ADI-R Repetitive Behavior Impairment |
| --- | --- | --- | --- | --- |
| Best fitting Model | Model 3 | Model 1 | Model 3 | Model 4 |
| Fixed Effects |  |  |  |  |
| Sex (Female=1) | -.40 (.55) | -.32 (.23) | -.14 (.39) | -.03 (.22) |
| T1 age | .21 (.11) | .06 (.02)* | .17 (.08)* | .41 (.10)*** |
| ID (ID = 1) | 3.41 (1.1)** | 1.13 (.22)*** | 2.50 (.80)*** | 1.11 (.65) |
| Age | -.22 (.05)*** | .004 (.013) | -.21 (.04)*** | -.46 (.07)*** |
| Age-squared | -- | -- | -- | .01 (.00)*** |
| Age x ID | .30 (.07)*** | -- | .19 (.05)** | .38 (.09)*** |
| Age-sq x ID | -- | -- | -- | -.016 (.004)*** |
| Age x T1 age | .00 (.00) | -.00 (.00) | .00 (.00) | -.01 (.01) |
| Age-sq x T1 age | -- | -- | -- | -.00 (.00) |
| ID x T1 age | -.07 (.13) | -- | -.11 (.10) | -.43 (.13) |
| Age x ID x T1 age | **-.00 (.00)** | -- | **-.00 (.00)** | .01 (.01) |
| Age-sq x ID x T1 age | -- | -- | -- | **.00 (.00)** |
| Constant | 12.1 (.85)*** | 3.70 (.25)*** | 8.38 (.58)*** | 5.14 (.49)*** |
| Random Effects |  |  |  |  |
| Var. (Age) | .031 [.016, .062] | -- | .013 [.005, .034] | .003 [.001, .014] |
| Var. (intercept) | 20.4 [15.5, 26.7] | 3.49 [2.98, 4.08] | 8.92 [6.39, 12.5] | 3.91 [2.81, 5.46] |
| Cov. (Age, intercept) | -.243 [-.531, .014] | -- | -.134 [-.302, .033] | -.058 [-.131, .016] |
| Var. (Level-1 residual) | 7.43 [6.81, 8.10] | 1.73 [1.60, 1.87] | 4.26 [3.85, 4.71] | 2.67 [2.46, 2.90] |

* p < .05; ** p < 0.01; *** p < 0.001

a. Estimated variances (Var.) and covariances (Cov.) of random parts of the mixed models are reported with 95% confidence intervals in brackets.

note: *ID* Intellectual Disability, *ADI-R* Autism Diagnostic Interview-Revised.

Table S2.2. Effects of Time 1 Age on the Best Fitting Growth Curve Models for Behavioral Functioning Measures.

|  | S2.2A. Activities of Daily Living (W-ADL) | S2.2B. Maladaptive Behavior (SIB-R total score) | S2.2C. Time Spent with Friends/Neighbors |
| --- | --- | --- | --- |
| Best fitting Model | Model 4 | Model 2 | Model 2 |
| Fixed Effects |  |  |  |
| Sex (Female=1) | -.57 (.62) | 1.70 (.88) | .26 (.12)* |
| T1 age | .24 (.18) | .27 (.19) | .06 (.03)* |
| ID (ID = 1) | -5.14 (1.4)*** | 5.77 (.88)*** | -.61 (.12)*** |
| Age | .87 (.08)*** | -.79 (.11)*** | .05 (.02)** |
| Age-squared | -.02 (.00)*** | .02 (.00)*** | -.002 (.001)*** |
| Age x ID | -.61 (.11)*** | -- | -- |
| Age-sq x ID | .01 (.00)** | -- | -- |
| Age x T1 age | -.03 (.01)** | -.01 (.01) | .00 (.00) |
| Age-sq x T1 age | -.001 (.000)*** | **-.00 (.00)** | **.00 (.00)** |
| ID x T1 age | .08 (.21) | -- | -- |
| Age x ID x T1 age | .01 (.01) | -- | -- |
| Age-sq x ID x T1 age | **-.0004 (.0002)*** | -- | -- |
| Constant | 19.4 (1.05)*** | 115.1 (1.4)*** | 1.54 (.18)*** |
| Random Effects |  |  |  |
| Var. (Age) | .032 [.023, .045] | .157 [.117, .212] | .002 [.001, .003] |
| Var. (intercept) | 25.0 [19.9, 31.5] | 112.5 [91.6, 138.2] | 1.11 [.806, 1.52] |
| Cov. (Age, intercept) | -.212 [-.416, -.008] | -3.31 [-4.25, -2.36] | -.022 [-.037, -.007] |
| Var. (Level-1 residual) | 6.09 [5.58, 6.64] | 33.6 [31.4, 35.9] | .973 [.906, 1.04] |

* p < .05; ** p < 0.01; *** p < 0.001

a. Estimated variances (Var.) and covariances (Cov.) of random parts of the mixed models are reported with 95% confidence intervals in brackets.

note: *ID* Intellectual Disability, *W-ADL* Waisman Activities of Daily Living Scale, *SIB-R* Scales of Independent Behaviors-Revised.

Table S2.3. Effects of Time 1 Age on the Best Fitting Growth Curve Models for Health Measures.

|  | S2.3A. Health Rating | S2.3B. # Medications: Psychotropic | S2.3C. # Medications: Non-psychotropic |
| --- | --- | --- | --- |
| Best fitting Model | Model 1 | Model 3 | Model 4 |
| Fixed Effects |  |  |  |
| Sex (Female=1) | -.08 (.06) | -.10 (.12) | .22 (.11)* |
| T1 age | .00 (.01) | -.03 (.02) | .03 (.04) |
| ID (ID = 1) | .06 (.06) | .05 (.22) | .33 (.28) |
| Age | -.02 (.00)*** | .02 (.01) | .03 (.03) |
| Age-squared | -- | -- | .00 (.00) |
| Age x ID | -- | .04 (.01)** | .09 (.03)** |
| Age-sq x ID | -- | -- | -.00 (.00) |
| Age x T1 age | **.00 (.00)** | .00 (.00) | -.01 (.00)* |
| Age-sq x T1 age | -- | -- | .0001 (.0000)** |
| ID x T1 age | -- | -.01 (.02) | -.13 (.05)** |
| Age x ID x T1 age | -- | **-.00 (.00)** | .01 (.00) |
| Age-sq x ID x T1 age | -- | -- | **-.00 (.00)** |
| Constant | 3.29 (.07)*** | .84 (.18)*** | .23 (.21) |
| Random Effects |  |  |  |
| Var. (Age) | .000 [.000, .001] | .003 [.003, .004] | .003 [.003, .005] |
| Var. (intercept) | .282 [.210, .378] | 1.09 [.868, 1.37] | .657 [.449, .960] |
| Cov. (Age, intercept) | -.005 [-.008, -.001] | -.030 [-.042, -.017] | -.020 [-.034, -.007] |
| Var. (Level-1 residual) | .246 [.230, .263] | .406 [.379, .435] | .762 [.712, .815] |

* p < .05; ** p < 0.01; *** p < 0.001

a. Estimated variances (Var.) and covariances (Cov.) of random parts of the mixed models are reported with 95% confidence intervals in brackets.

note: *ID* Intellectual Disability.
